# Ocular side effects of novel anti-cancer biological therapies

**DOI:** 10.1038/s41598-020-80898-7

**Published:** 2021-01-12

**Authors:** Vicktoria Vishnevskia-Dai, Lihi Rozner, Raanan Berger, Ziv Jaron, Sivan Elyashiv, Gal Markel, Ofira Zloto

**Affiliations:** 1 The Ocular Oncology Service, The Goldschleger Eye Institute, Sheba Medical Center, Tel Hashomer, Israel; 2 Institute of Oncology, Sheba Medical Center, Tel Hashomer, Israel; 3 Sackler Faculty of Medicine, Tel Aviv University, Tel Aviv, Israel

**Keywords:** Diseases, Medical research, Oncology

## Abstract

To examine the ocular side effects of selected biological anti-cancer therapies and the ocular and systemic prognosis of patients receiving them. We retrospectively reviewed all medical records of patients who received biological anti-cancer treatment from 1/2012 to 12/2017 and who were treated at our ocular oncology service. The following data was retrieved: primary malignancy, metastasis, type of biological therapy, ocular side effects, ophthalmic treatment, non-ocular side effects, and ocular and systemic disease prognoses. Twenty-two patients received biological therapies and reported ocular side effects. Eighteen patients (81.8%) had bilateral ocular side effects, including uveitis (40.9%), dry eye (22.7%), and central serous retinopathy (22.7%). One patient (4.5%) had central retinal artery occlusion (CRAO), and one patient (4.5%) had branch retinal vein occlusion (BRVO). At the end of follow-up, 6 patients (27.27%) had resolution of the ocular disease, 13 patients (59.09%) had stable ocular disease, and 3 patients (13.64%) had progression of the ocular disease. Visual acuity improved significantly at the end of follow-up compared to initial values. Eighteen patients (81.8%) were alive at study closure. Biological therapies can cause a wide range of ocular side effects ranging from dry eye symptoms to severe pathologies that may cause ocular morbidity and vision loss, such as uveitis, CRAO and BRVO. All patients receiving biological treatments should be screened by ophthalmologists before treatment, re-screened every 4–6 months during treatment, and again at the end of treatment. Patients on biological treatment who have ocular complaints should be urgently referred to ocular consultation for early identification and early intervention.

## Introduction

Cancer is the leading cause of death in the developed world with a mortality case of over 10 million mortality cases annually^1^. The traditional non-surgical treatments for cancer are radiation and chemotherapeutic drugs. However, those treatments also affect healthy cells, causing numerous side effects, some of which lead to severe morbidity^2^. Therefore, the current trend is focused on finding targeted therapies that eliminate specifically cancerous cells only. In the last 2 decades, studies on the molecular basis, epigenetic changes, and gene expression in cancer, as well as new diagnostic technologies have led to advances in understanding the mechanism of cancer development and the discovery of new modalities of therapy^3,4^. One of these novel modalities used for various cancer lines is biological therapy. Biological therapy stimulates the body’s own immune system to act against cancer cells or interfere with tumor growth and progression by specific molecules or antibodies^5–7^. The different types of biological therapies include immune checkpoint inhibitors, immune cell therapy, therapeutic antibodies/immune system molecules, therapeutic vaccines, and immune system modulators^8^. Although those treatments are targeted and may effectively control tumor growth, they still may have side effects in the digestive system, liver, skin, nervous system, heart, and more^9^. Very few studies examined the ocular side effects of those treatments, and most of them were conducted on small groups and focused on a specific medication^10–12^.

The purposes of the current study is to examine the ocular side effects of various biological therapies as well as to examine ocular and systemic prognoses of the patients receiving them. This knowledge may help individualize patient management and lead to improved vision and quality of life.

## Methods

### Patients

The medical records of all consecutive patients with ocular side effects while receiving biological anti-cancer treatment who presented to the Ocular Oncology Service of the Goldschleger Eye Institute from January 2012 to December 2017 were retrospectively reviewed. The retrieved data included demographics, primary malignancy, metastasis status, type of biological therapy, laterality of the ocular side effects, ocular side effects, ophthalmic examination, ophthalmic treatment, non-ocular side effects, and both the ocular and systemic disease prognoses. The biological treatments were divided into the following groups according to their mechanisms of action:

- *Group 1* Molecularly targeted therapies—BRAF inhibitors (Vemurafenib) + MEK inhibitors (Trametinib, Pimasertib)
- *Group 2* Immune checkpoint inhibitor—Cytotoxic T-lymphocyte antigen-4 (CTLA-4, Ipilimumab), Programmed death protein 1 (PD-1, Pembrolizumab, Nivolumab), and Programmed death ligand-1 (PD-L1, Durvalumab)
- *Group 3* Therapeutic Antibodies/Immune System Molecule—anti-epidermal growth factor receptor (EGFR inhibitor) + anaplastic lymphoma kinase (ALK, Alectinib)
- *Group 4* Other—Bacillus Calmette-Guerin, Ibrutinib, Ixazomib, Pemetrexed

The visual acuity (VA) was examined with Snellen VA charts and converted to log minimum angle of resolution (Log MAR) values at the beginning and end of follow-up. The ocular examination included slit-lamp and fundus examinations. Ancillary imaging testing [ultrasound (US), optical coherence tomography (OCT), fundus photos, and others] were performed as indicated. The treatment modalities varied according to the ocular pathology—for example: for dry eye patients were treated by lubricants, for anterior uveitis by topical steroids and pupil dilator and for posterior and panuveitis by topical steroids or steroid injection depend on the severity of the disease.

This retrospective interventional cohort study was approved by the local institutional review board (IRB) of Sheba Medical Center, which waived informed consent. All methods were performed in accordance with to National Institutes of Health guidelines of Israel.

### Statistical analysis

Quantitative variables were described as mean, range, and standard deviation. Categorical variables were described as absolute and relative frequencies. Paired t-test analyses compared VA at presentation and at the end of follow-up. The overall significance level was set to an alpha of 0.05. The statistical analysis was carried out with Microsoft Excel 2017 (Microsoft Corporation, Redmond, WA) and IBM SPSS software version 24.0 (SPSS, Inc., Chicago, IL, USA).

## Results

### Demographics

Between January 2012 and December 2017, a total of 22 patients (11 men and 11 women) were treated by biological therapies and reported ocular side effects. Their mean age at diagnosis was 63.32 ± 15.17 years (range 29–89 years).

### Ocular history

Eleven patients (50%) had undergone cataract surgery, one patient (4.5%) had central serous retinopathy (CSR), and one patient (4.5%) had an epiretinal membrane (ERM) before embarking upon the biological treatment. All those patients had the ocular surgeries or problems at least one year before starting the biological treatment. None of the patients had a history of uveitis.

### Characteristics of the primary malignancy

Ten patients (45.5%) had skin melanoma, 4 patients (18.2%) had non-small-cell lung carcinoma, 2 patients (2.8%) had transitional cell carcinoma, and one patient (1.2%) each had small-cell lung carcinoma, diffuse large B-cell lymphoma, uveal melanoma, cervix uteri adenosquamous carcinoma, chronic lymphocytic leukemia, and multiple myeloma. Nineteen patients (86.36%) had metastatic disease to the bone (10 patients, 45.5%), to the liver (7 patients, 31.8%), to the brain (6 patients, 27.3%), to the lung (6 patients, 27.3%), to the mesenteric fat (2 patients, 10.52%), to the pleura (2 patients,10.52%), and one patient (4.5%) each to the retroperitoneum, adrenal, omental fat, and breast. Three patients (13.64%) had stage 3 disease. The distribution of the biological therapies is summarized in Table 1. There were no demographic differences between the groups (Table 2).

**Table 1:** Biological therapies.

Type of treatment	Number of cases	Percent
**Name of biological drug**		
Ibrutinib	1	4.5
Alectinib	2	9.1
Bacillus Calmette–Guerin	1	4.5
Durvalumab	1	4.5
EGFR inhibitors	1	4.5
Ipilimumab	1	4.5
Ixazomib	1	4.5
Nivolomab	1	4.5
Pembrolizumab	2	9.1
Pemetrexed	1	4.5
Pimasertib	1	4.5
Trametinib	1	4.5
Vemurafenib	8	36.4
**Biological treatment groups**		
Anaplastic lymphoma kinase (ALK)	2	9.1
BRAF inhibitors	8	36.4
Cytotoxic T-lymphocyte antigen-4 (CTLA-4)	1	4.5
Epidermal growth factor receptor (EGFR)	1	
MEK1/2	2	4.5
Programmed death ligand-1 (PD-1)	4	9.1
Other	4	18.2
**Biological treatment groups by mechanisms**		
Group 1—BRAF + MEK	10	45.5
Group 2—CTLA4 + PD1	5	22.7
Group 3—EGFR + ALK	3	13.6
Group 4—Other	4	18.2
**Additional treatments—before biological treatment**		
Chemotherapy	6	27.3
Radiation	6	27.3
Surgery	6	27.3
**Systemic side effects**		
Musculoskeletal	6	27.3
Skin	13	59.1
Gastrointestinal	7	31.8
Others	12	54.5

**Table 2:** Demographic charistricts of the groups.

Biological treatment groups by mechanisms	Number of patients	Gender Male: female	Age (mean)
Group 1—BRAF + MEK	10	6:4	60.50
Group 2—CTLA4 + PD1	5	1:4	72.60
Group 3—EGFR + ALK	3	2:1	59.67
Group 4—Other	4	2:2	67.00
*p* value		0.469	0.499

### Ocular side effects

Eighteen patients (81.8%) had bilateral ocular side effects, 2 patients (9.1%) had side effects only to the right eye, and 2 patients (9.1%) had side effects only to the left eye. The side effects and ocular treatments are summarized in Table 3. There was no difference in ocular side effects and the various types of biological treatment mechanisms (*p* = 0.219 and *p* = 0.235, respectively, χ^2^). Table 4 lists the differences in ocular and side effect characteristics of each group. The mean pre-treatment VA of right eyes that had side effects was 0.974 ± 0.194 and 0.754 ± 0.481 (t-test, *p* = 0.046) post-treatment. The mean pre-treatment VA of left eyes that had side effects was 0.893 ± 0.146 and 0.650 ± 0.031 post-treatment (t-test, *p* = 0.016). There were no differences in VA at diagnosis or at the end of follow-up between the different groups of treatment (Table 5).

**Table 3:** Ocular side effects and treatment.

Variable	Number of cases	Percent
**Side effects**		
Uveitis (anterior, posterior, panuveitis)	9 (7, 1, 1)	40.9 (77.8, 11.1, 11.1)
Dry eye	5	22.7
CSR-like	5	22.7
Vitreitis	2	9.1
CRAO	1	4.5
CME	1	4.5
Trichomegaly	3	13.6
BRVO	1	4.5
**Treatment**		
Intravitreal bevacizumab injection	1	4.5
Intravitreal Kenalog injection	2	9.00
Topical		
Artificial tears	5	22.5
Steroids	9	40.5
Pupil dilator	7	31.5
Oral therapy—prednisone	9	40.5
Surgical—pars plana vitrectomy	1	4.5

CSR, central serous retinopathy; CRAO, central retinal artery occlusion; CME, cystoid macular edema; BRVO, branch retinal vein occlusion.

**Table 4:** Ocular and side effect characteristic of biological treatment mechanism groups.

Variable	Group 1 N (%)	Group 2 N (%)	Group 3 N (%)	Group 4 N (%)	*p* value (χ^2^)
**Past ocular history**					0.081
Cataract	4 (40)	5 (100)	1 (33.3)	1 (25)	
ERM	0 (0)	1 (20)	0 (0)	0 (0)	
CSR	0 (0)	0 (0)	1 (33.3)	0 (0)	
**Side effects**					0.219
posterior, panuveitis)	4 (40) (3, 0, 1)	3 (60) (2, 1, 0)	0 (0) (0, 0, 0)	2 (50) (2, 0, 0)	
CSR-like	3 (30)	(0)	1 (33.3)	1 (25)	
Dry eye	2 (20)	2 (40)	0 (0)	1 (12.5)	
Vitreitis	2 (20)	0 (0)	0 (0)	0 (0)	
CRAO	0 (0)	1 (20)	0 (0)	0 (0)	
CME	1 (10)	0 (0)	0 (0)	0 (0)	
Trichomegaly	0 (0)	0 (0)	2 (66.6)	1 (25)	
BRVO	0 (0	1 (20)	0 (0)	0 (0)	
**Treatment**					0.325
Intravitreal injection	2	1	0	0	
Topical	5	4	0	3	
Medical	1	0	0	0	
Surgical	1	0	0	0	

CSR, central serous retinopathy; CRAO, central retinal artery occlusion; CME, cystoid macular edema; BRVO, branch retinal vein occlusion.

**Table 5:** Visual acuity at diagnosis versus at the end of follow-up.

Variable	Group 1 Mean ± SD	Group 2 Mean ± SD	Group 3 Mean ± SD	Group 4 Mean ± SD	*p* value between groups
Visual acuity at diagnosis: OD	0.932 ± 0.150	1.00 ± 0.205	0.928 ± 0.212	1.100 ± 0.288	0.497
Visual acuity at the end of follow-up: OD	0.594 ± 0.534	1.00 ± 0.115	0.389 ± 0.550	1.086 ± 0.303	0.268
*p* value at diagnosis versus at the end of follow-up	0.091	0.981	< 0.01	0.391	
Visual acuity at diagnosis: OS	0.843 ± 0.071	1.00 ± 0.240	0.836 ± 0.101	0.945 ± 0.169	0.150
Visual acuity at the end of follow-up: OS	0.539 ± 0.484	0.846 ± 0.085	0.551 ± 0.379	0.912 ± 0.180	0.267
*p* value at diagnosis versus at the end of follow-up	0.072	0.163	0.064	0.391	

### Prognosis

The mean duration of follow-up was 20.88 ± 40.69 months (range 1–177). The short follow-up of one month was that of a single patient who died of the primary systemic disease. The biological treatment was stopped in 9 patients (40.91%), 4 because of systemic and not ocular side effects and the other 5 because of improvement in their disease. Ocular treatment was not stopped in any of the patients. At the end of follow-up, 6 patients (27.27%) had resolution of the ocular disease, 13 patients (59.09%) had stable ocular disease, and 3 patients (13.64%) had progression of the ocular disease. There was no differences in ocular prognosis between the different groups of the biological therapies (*p* = 0.187, χ^2^). At the end of the study period, 18 patients (81.8%) were alive: 11 of them (50%) had stable systemic disease, and 7 (31.8%) had systemic progressive disease.

## Discussion

Novel types of biological therapies, particularly immunotherapy, have had a marked impact on cancer patient survival, and they are now more commonly implemented for various types of cancer^13^. Although biological therapy is generally less toxic to normal cells compared to chemotherapy, biological treatments can lead to numerous systemic side effects to the skin, joints, heart, lungs, liver, kidneys, central nervous system, etc. The most common side effects are: fatigue (26–53%), skin rash (1–50%), lymphocytopenia (10–49%), and increased pathological liver function tests (1–46%)^14–16^.

Ocular side effects of immunotherapy are considered uncommon, occurring in approximately 1% of patients while from chemotherapy in various reports the ocular side effects are higher than 1%^17–19^. They can affect various parts of the eye and orbit^20,21^. The most commonly reported ocular side effects of immunotherapy are dry eye (1–24%), inflammatory uveitis (1%), and myasthenia gravis with ocular involvement^10,20^. In this study, we report 22 patients that were treated with biological therapy for advanced or metastatic cancer and referred to our ocular oncology service due to ocular complaints. The most common side effect was inflammatory uveitis (40.9%), and 13 of the affected patients (59%) were treated with topical or systemic corticosteroid without cessation of the biological treatment. All 22 patients showed improvement in their inflammation reaction as well as improvement of their complaints and vision. One patient with posterior uveitis and one patient with panuveitis who were treated with injections of corticosteroid also showed clinical improvement.

Other common side effects were dry eye syndrome and CSR-like reaction. Dry eye is considered the most common side effect of biological treatments on the ocular anterior segment^22,23^. The reported severity of the symptoms were variable. Nguyen et al. reported one case of corneal perforation due to dry eye after biological treatment^23^. The patients with dry eyes in our cohort were treated locally with artificial preservative free tears and topical cyclosporine (1%), with improvement in their symptoms and none with severity that led to corneal perforation. Another common side effect was CSR-like reaction with subretinal fluid formation. This reaction had also been described after biological therapy with anthrax vaccination^24^. In our cohort, none of the patients required treatment for CSR-like reaction. The subretinal fluid was absorbed and the condition resolved without intervention in all cases.

Two patients in our cohort developed severe ocular side effects after biological treatment, specifically, CRAO and BRVO. Those patients were 67 and 90 years old, respectively with positive medical history for vascular risk factors (hypertension and hyperlipidemia) without history of diabetic mellites or stroke. In general, since patients with CRAO experience severe painless loss of vision and are also at increased risk for stroke^25^, it is recommended that treatment with anti-thrombotic drugs be considered^26^. Patients with BRVO are at increased risk of developing macular edema and vision loss^27^. Although CRAO and BRVO are uncommon side effects, their grave impact on vision warrants heightened awareness and urgent screening in patients with ocular side effects during or after biological treatments.

To the best of our knowledge, this is the first report on comparative ocular side effects in various biological treatment groups. Analysis of our findings failed to reveal any such differences among our study patients, although that may be attributed to the small number of patients in this study. To date, there are no published guidelines for ophthalmic examinations before, during, and after biological therapies. The current study results demonstrated that biological therapies can cause ocular discomfort due to dry eye symptoms. Moreover, sever pathologies like uveitis, CRVO and BRVO can occur and lead to severe eye morbidity. Therefore, we believe that all patients who start biological treatments should be screened by ophthalmologists before treatment, re-screened every 4–6 months during the treatments, and again at the end of the treatment. Any patient on biological treatment who presents with ocular complaints should be urgently referred to ocular consultation. Early identification of the ocular side effects of cancer therapy may lead to better visual prognosis.

The limitations of this study are its retrospective nature, the small number of patients and no standardized diagnosis and periodical follow up protocol. We speculate that ocular side effects after biological treatment are more common than our findings, and that the unreported cases may have less severe symptoms for which they are treated at community clinics. This may explain our small number of patients and may cause a referral bias of only the more severe cases to our ocular oncology service. A larger study with planned screening of all patients that are treated with biological treatment at one center with a periodical ocular follow-up protocol (every 4–6 months) should be performed to better assess the prevalence of ocular side effects of biological treatment. To note in our cohort in 20/22 patients (90.9%) the ocular side effects were successfully controlled and in none of the cases the ocular side effects lead to biological treatment cessation. This fact is of most importance since these treatments can substantially improve patient’s survivor.

In summary, we present a cohort of 22 patients who received various biological treatments for advanced or metastatic cancer and developed ocular side effects. The most common side effects were uveitis, dry eye syndrome and CSR-like reaction,. Some severe side effects, such as BRVO and CRAO, were also reported. The patients were treated according to their ocular diagnosis, with improvement in their VA and ocular symptoms. There was no case of treatment cessation because of ocular side effects. Larger studies are required in order to examine the prevalence of ocular side effects and the differences of their occurrence between the various groups of biological treatments as well as to compare those side effects to chemotherapy and radiotherapy.

## Abbreviations

ALK: Anaplastic lymphoma kinase
BRAO: Branch retinal artery occlusion
CTLA-4: Cytotoxic T-lymphocyte antigen-4
CRAO: Central retinal artery occlusion
EGFR: Epidermal growth factor receptor
Log MAR: Log minimum angle of resolution
MEK: Mitogen-activated protein kinase
OCT: Optical coherence tomography
PD-1: Programmed death protein 1
PD-L1: Programmed death ligand-1
RAF: Rapidly accelerated fibrosarcoma kinase
US: Ultrasound
VA: Visual acuity
